# Keeping phase III tuberculosis trials relevant: Adapting to a rapidly changing landscape

**DOI:** 10.1371/journal.pmed.1002767

**Published:** 2019-03-22

**Authors:** Patrick P. J. Phillips, Carole D. Mitnick, James D. Neaton, Payam Nahid, Christian Lienhardt, Andrew J. Nunn

**Affiliations:** 1 School of Medicine, UCSF Center for TB, University of California San Francisco, San Francisco, California, United States of America; 2 Harvard Medical School, Boston, Massachusetts, United States of America; 3 School of Public Health, University of Minnesota, Minneapolis, Minnesota, United States of America; 4 Unité Mixte Internationale TransVIHMI (UMI 233 IRD, U1175 INSERM, Université de Montpellier), Institut de Recherche pour le Développement Montpellier, Montpellier, France; 5 Medical Research Council Clinical Trials Unit at University College London, London, United Kingdom

## Abstract

In a Collection Review, Patrick Phillips and colleagues discuss developments in clinical trial design for the evaluation of TB therapeutics.

**Summary points**

- The landscape of tuberculosis (TB) treatment has evolved considerably over the last 10 years, necessitating careful consideration of various trial design aspects to ensure that TB phase III trials are still impactful at trial completion, often more than 4–5 years after initial design.
- The choice of control is guided by the specific trial objectives, weighing the relative merits of internal validity and external generalizability alongside randomization in making the correct inference. A particular challenge occurs when international or national guidelines change during the trial.
- Improved execution and relevance of noninferiority trials for TB require greater emphasis on study quality, especially maximizing treatment adherence and minimizing missing outcome data; preferred use of intention-to-treat rather than per-protocol analyses; more careful justification of the margin of noninferiority; and consideration of recent innovations such as a Bayesian approach to noninferiority.
- Many adaptive trial designs are well suited to optimization of TB treatment. A thorough understanding of type I error rates and biases in treatment effect estimates is critical for regulatory approval and consideration in establishing World Health Organization (WHO) guidelines.
- Treatment stratification is an area of limited experience for TB trials, and trialists must learn from well-established methodology in other disease areas.
- Explanatory trials are important for evaluating the efficacy of an intervention under close to ideal conditions. However, no single trial can address all relevant questions about a given therapeutic intervention at one time, and pragmatic trials will be essential for public health and policy decision-making purposes.
- TB treatment trials today should favor bold and creative approaches that can produce high-quality evidence for effective, patient-centered care made accessible to all 10 million new TB patients, including the half-million with drug-resistant TB (DR-TB), each year.

## Introduction

One of the first multicenter randomized trials was the British Medical Research Council (MRC) streptomycin trial [1]. From the first meeting of the special committee to “plan trials of streptomycin in tuberculosis” in September, 1946, the primary trial results from 107 participants followed for 12 months were published in the *British Medical Journal* two years later in October, 1948 [2]. Although treatment with a single drug was subsequently shown to be inadequate because of the generation of drug resistance [3], the results of the trial changed clinical practice [1].

It has, however, become difficult to conduct phase III clinical trials in the 21st century in any disease area in such a short time frame. Trials often require more patients to show benefit, and their initiation is often protracted because of the need for independent ethical review(s), approval by national regulatory bodies, and the training and compliance with Good Clinical Practice (GCP) that is necessary to ensure that the trial is designed and conducted to the highest standards. All of these changes have been aimed, rightly, at protecting participants and ensuring reliable results, but they have also limited the ability to conduct clinical trials to respond quickly to important public health questions, especially in the context of a rapidly evolving disease and treatment landscape. The interval from start of enrollment to first public presentation of results of recent phase III tuberculosis (TB) trials ranges from 4.6–8.4 years [4–7]. This does not include time for design, planning, and ethical and regulatory approvals prior to start of recruitment, which commonly takes at least a year, and is consistent with a systematic review of time to publication of results across other disease areas [8].

The landscape of TB treatment has evolved considerably over the last 10 years—particularly in the management of drug-resistant TB (DR-TB). Changes include the earlier diagnosis of DR-TB with widespread implementation of newer tests such as GeneXpert [9,10] and Line Probe Assays [11], a better understanding of the pharmacology and bactericidal activity of the various drugs used [12], and the introduction of new drugs (bedaquiline with accelerated approval by the United States Food and Drug Administration [FDA] in December, 2012 and delamanid with conditional approval by the European Medicines Agency [EMA] in November, 2013), as well as observational studies and clinical trials investigating various combinations of current, new, and repurposed drugs in an attempt to shorten DR-TB therapies [13–17]. These developments are reflected in evolving World Health Organization (WHO) guidance for DR-TB (see **Table 1**). Furthermore, knowledge about the epidemic itself continues to evolve with a recognition of the growing importance of the transmission of DR-TB [18,19] and increasing levels of second-line drug resistance [20]. Thus, in 2017, among the 10.0 million people developing TB disease, 558,000 (5.6%) developed a form that was resistant to at least rifampicin, the most effective first-line drug, and 230,000 died of it. The severity of national epidemics varies widely among countries. Estimated prevalence of rifampicin-resistant TB (RR-TB) among new TB cases ranges from 1.3% in Kenya to 38.0% in Belarus among the 30 high-TB–burden countries [20].

**Table 1:** Summary of WHO guidelines, policies, and statements on the treatment of DR-TB. Guidelines for the treatment of DS-TB are not included because these have remained largely unchanged in this period. Key dates are also included from the case study of the STREAM trial, a trial comparing a 9- to 11-month regimen containing high-dose moxifloxacin and clofazimine with the 20- to 24-month WHO-recommended standard of care regimen for MDR-TB, which is discussed further in **Box 1**.

Key event in STREAM trial	Date of publication or event	WHO document title
	1996	Guidelines for the management of multidrug-resistant tuberculosis
	2000	Guidelines for establishing DOTS-Plus pilot projects for the management of multidrug-resistant tuberculosis (MDR-TB)
	2006	Guidelines for the programmatic management of drug-resistant tuberculosis
	2008	Guidelines for the programmatic management of drug-resistant tuberculosis. Emergency Update 2008
	June, 2011	Guidelines for the programmatic management of drug-resistant tuberculosis. 2011 update
First participant enrolled in STREAM Stage 1 trial	July, 2012	
	June, 2013	The use of bedaquiline in the treatment of multidrug-resistant tuberculosis: Interim policy guidance
	October, 2014	The use of delamanid in the treatment of multidrug-resistant tuberculosis: Interim policy guidance
Last participant enrolled in STREAM Stage 1 trial	June, 2015	
First participant enrolled in STREAM Stage 2 trial	April, 2016	
	May, 2016	WHO treatment guidelines for drug-resistant tuberculosis. 2016 update
	October, 2016	WHO treatment guidelines for drug-resistant tuberculosis. 2016 update. October 2016 revision
	October, 2016	The use of delamanid in the treatment of multidrug-resistant tuberculosis in children and adolescents: Interim policy guidance
	March, 2017	Report of the Guideline Development Group Meeting on the use of bedaquiline in the treatment of multidrug-resistant tuberculosis. A review of available evidence (2016)
Preliminary results from STREAM Stage 1 trial presented at 48th Union Conference on Lung Health	October, 2017	
	January, 2018	WHO position statement on the use of delamanid for multidrug-resistant tuberculosis
	March, 2018	WHO treatment guidelines for isoniazid-resistant tuberculosis: Supplement to the WHO treatment guidelines for drug-resistant tuberculosis
	April, 2018	Position statement on the continued use of the shorter MDR-TB regimen following an expedited review of the STREAM Stage 1 preliminary results
	August, 2018	Rapid Communication: Key changes to treatment of multidrug- and rifampicin-resistant tuberculosis (MDR/RR-TB)
Final results from STREAM Stage 1 trial presented at 49th Union Conference on Lung Health	October, 2018	
	December, 2018	WHO treatment guidelines for multidrug- and rifampicin-resistant tuberculosis. 2018 update. Pre-final text
	Expected early 2019	WHO treatment guidelines for multidrug- and rifampicin-resistant tuberculosis. Final text

**Abbreviations**: DS-TB, drug-sensitive tuberculosis; MDR-TB, multidrug-resistant tuberculosis; RR-TB, rifampicin-resistant tuberculosis; TB, tuberculosis; WHO, World Health Organization.

Given the unavoidably protracted duration of phase III TB trials in the 21st century, the status of TB as a global health priority (the first ever United Nations [UN] General Assembly high-level meeting on TB was held in September, 2018), and the increasing trial costs relative to a huge shortfall in research and development funding [21], those who conduct clinical trials are obligated to design them in such a way that they will have a direct impact on policy and practice of TB treatment at the projected time of trial completion. In this paper, as part of a *PLOS Medicine* Collection on Advances in Clinical Trial Design for Development of New TB Treatments [22], we discuss how phase III TB trials could be designed with such “future-proofing” in mind.

## Choice of control

It is usual for the comparator in a clinical trial to be the standard of care treatment so that the results can be interpreted in relation to current practice [23]. Furthermore, the principle of clinical equipoise provides an ethical obligation to ensure patients on the control arm receive the best available standard of care [24]. In drug-sensitive TB (DS-TB), a 6-month regimen of rifampicin and isoniazid, supplemented by pyrazinamide and ethambutol in the first 2 months, is the recognized standard of care [25]; all recent phase III trials have therefore used this regimen as the internal control. For DR-TB, WHO guidelines provide a recipe for constructing an effective regimen based on the combination of drugs from various classes, leading to variability in terms of regimen composition across patients and trial sites. The most recent guidelines go further and recommend both long and short regimens [26]. For these reasons, and in the absence of an established standard, trials have selected various approaches to the choice of control (see **Table 2** for a discussion of advantages and limitations). For example, the design that adds a single new drug (or placebo) to a background regimen has shown its limitations because it provides no information on the optimal regimen within which the new drug can be used. The specific trial objectives will guide the choice of control, weighing the relative merits of internal validity and external generalizability alongside randomization in making the correct inference [27]. In any case, the implications of each approach on the trial’s statistical considerations as well as the final interpretation of the trial results need to be carefully considered from the outset.

**Table 2:** Controls used in DR-TB trials.

Choice of control	Examples	Strengths	Limitations
Placebo, added to optimized background regimen	Delamanid phase II and III trials [28,29] Bedaquiline phase II trial [30,31], Opti-Q [32]	Permits blinding of healthcare providers and participants, yielding unbiased estimates of the efficacy and safety of the individual drug. This design was used to inform regulatory approval of new drugs.	Yields little or no information on how to use the drug in a regimen, which is essential for programmatic implementation; effect of drug can be masked if background regimen is highly effective.
External control (historical or concurrent)	NiX-TB (NCT02333799), ZeNiX-TB (NCT03086486)	Smaller sample size and operational efficiencies due to absence of randomization and use of only one regimen. Considered the only option if there is no accepted standard of care. The justification for use of a historic control can only be used in the first successful trial in that patient population; subsequent trials could use the previous intervention as internal control.	Highly dependent on choice of external control, differences between patient populations and secular trends (with a historical control) affect interpretation of results. Challenging to quantify how much “supportive care” in the trial affected outcomes relative to control outside trial [33].
Randomized comparison in DS-TB, parallel uncontrolled DR-TB cohort with same regimen	STAND (NCT02342886), SimpliciTB (NCT03338621)	Randomized comparison in DS-TB provides strong evidence for safety of regimen in TB patients and efficacy in DS-TB. The parallel DR-TB cohort informs whether results differ between the two TB patient populations.	Only appropriate for regimens that are targeted for both DS- and DR-TB. Extrapolation from DS-TB comparison to DR-TB population requires assumptions.
Local standard of care (varying by site)	STREAM Stage 1 [34], endTB [35]	Better external validity because of randomization to genuine standard of care, operational efficiencies because sites use local standard for control arm participants.	Control regimen may differ by site and over time. This will increase variability in results and may need to be accounted for by increasing sample size.
Prescriptive regimen	NEXT (NCT02454205), STREAM Stage 2 [36]	Better internal validity because of clear randomized comparison of two regimens.	Limited external validity since choice of control regimen may not reflect standard in many places. This would change if a standardized regimen is widely adopted; there are currently variations in how the short regimen is used (for example, choice of fluoroquinolone and bedaquiline in South Africa).

**Abbreviations**: DR-TB, drug-resistant TB; DS-TB, drug-sensitive TB; TB, tuberculosis.

An added complication arises when international or national guidelines change during the course of a trial, as exemplified in the STREAM trial [34,36] (see Box 1). If significant new developments arise during the course of a trial that may impact a participant’s willingness to continue, investigators have a responsibility to inform patients; this is part of the Federal Code of Regulations in the US. It may not be feasible or ethical to continue the trial without modification under such circumstances; conversely, if the evidence base for change is weak [37] and randomization among treatment arms is still possible, no change in the trial design may be warranted [38]. The investigators, usually with advice of an independent group such as the data and safety monitoring board (DSMB) or a community advisory group, should evaluate the new information and make a judgment about whether the trial protocol should be modified and how, if at all, participants should be informed. Policy makers and guideline developers can help with this by including explicit wording that further research is still needed when making recommendations that are based on low certainty in the evidence.

**Box 1. A case study: The STREAM trial [34,36]**

The STREAM trial was initiated to evaluate a novel 9- to 11-month regimen for the treatment of multidrug-resistant TB (MDR-TB) based on results from an observational cohort study in Bangladesh [13,16]. The primary objective of Stage 1 of this multicenter randomized trial was to determine whether a slightly modified version of this regimen (with high-dose moxifloxacin replacing gatifloxacin) was safe and at least as effective as the recommended standard of care. The first trial participant was enrolled in July, 2012, with the first results due to be published in 2019. During the trial period, the landscape changed substantially (as described in main text), and the trial had to adapt in a number of ways.

**Incorporating new drugs in additional trial arms**

The availability of new drugs without data on how to use them in combination regimens prompted the investigators to consider transitioning from a two-arm study (STREAM Stage 1) to a four-arm study (STREAM Stage 2), with the aim that any new arm(s) added to the trial should be shorter or simpler to take and intended to be less toxic. Through wide consultation, an injectable-sparing regimen to avoid the hearing loss associated with aminoglycoside use was selected. There was also preference for a regimen that excluded prothionamide and ethionamide because these drugs cause nausea and vomiting that compromise the tolerability of any MDR-TB regimen. The decision was therefore made to add four arms, a 9-month completely oral regimen in which kanamycin was replaced by bedaquiline and a shorter 6-month regimen in which bedaquiline replaced prothionamide and kanamycin duration was reduced to 8 weeks. The primary objective of Stage 2 was to evaluate whether the bedaquiline-containing regimens were safe with efficacy not inferior to that of the 9- to 11-month control regimen.

The first patient in Stage 2 was randomized in April, 2016. In order to ensure timely completion of the main comparison of the fully oral regimen with the 9- to 11-month injectable-containing regimen and the increasing desirability of an injection-free regimen, it was decided to terminate enrollment to the 6-month injectable-containing regimen in 2018.

**Choice of control**

The locally used 20- to 24-month regimen consistent with 2011 WHO guidelines [39] was selected as the control arm in Stage 1. Although results from Stage 1 were not available at the time that Stage 2 was initiated, the STREAM investigators took the unconventional step of including two control regimens: (i) the 9- to 11-month regimen studied as the intervention in Stage 1 and (ii) the locally used WHO-recommended regimen that had been the control in Stage 1. The second control was considered as a “reserve internal control.” Although it was not included in the primary objective and only 1 in 7 participants were to be allocated to this arm, it was to be used as a comparator in secondary analyses to permit interpretation of the results of the trial as compared to 2011 WHO guidelines.

In May, 2016, the revised MDR-TB treatment guidelines from WHO recommended a short regimen very similar to that being evaluated in the STREAM trial (see **Table 1**) for patients who met specific inclusion criteria. Subsequently, countries adopting these revised guidelines were no longer able to enroll patients to the second control arm. The protocol was therefore amended to exclude enrollment to the “reserve internal control” in these countries, thereby unfortunately reducing the value of comparisons to that regimen because of fewer participants.

Continued evolution in WHO guidance [26], and its implications for the control arm, is under consideration by the investigators.

**Balancing program and regulatory objectives**

The primary aim of the trial is to evaluate the efficacy and safety of new regimens for MDR-TB. The 9- to 11-month all-oral regimen in STREAM Stage 2 is the same as the 9- to 11-month control, except that kanamycin is replaced with bedaquiline. The comparison of these two regimens is therefore a randomized comparison of bedaquiline to kanamycin within a multidrug combination.

This is quite different from the add-on trial with the placebo comparison used in the pivotal phase II trial of bedaquiline [31,40] (see the first row in **Table 2**) because it provides evidence both of the efficacy and safety of new standardized regimens that include bedaquiline as well as of the long-term efficacy and safety of bedaquiline, albeit in comparison to kanamycin rather than placebo. With this, it has become the confirmatory phase III trial to be considered by the FDA following the accelerated approval of bedaquiline.

## Noninferiority, analysis populations, and “estimands”

**Fig 1:**
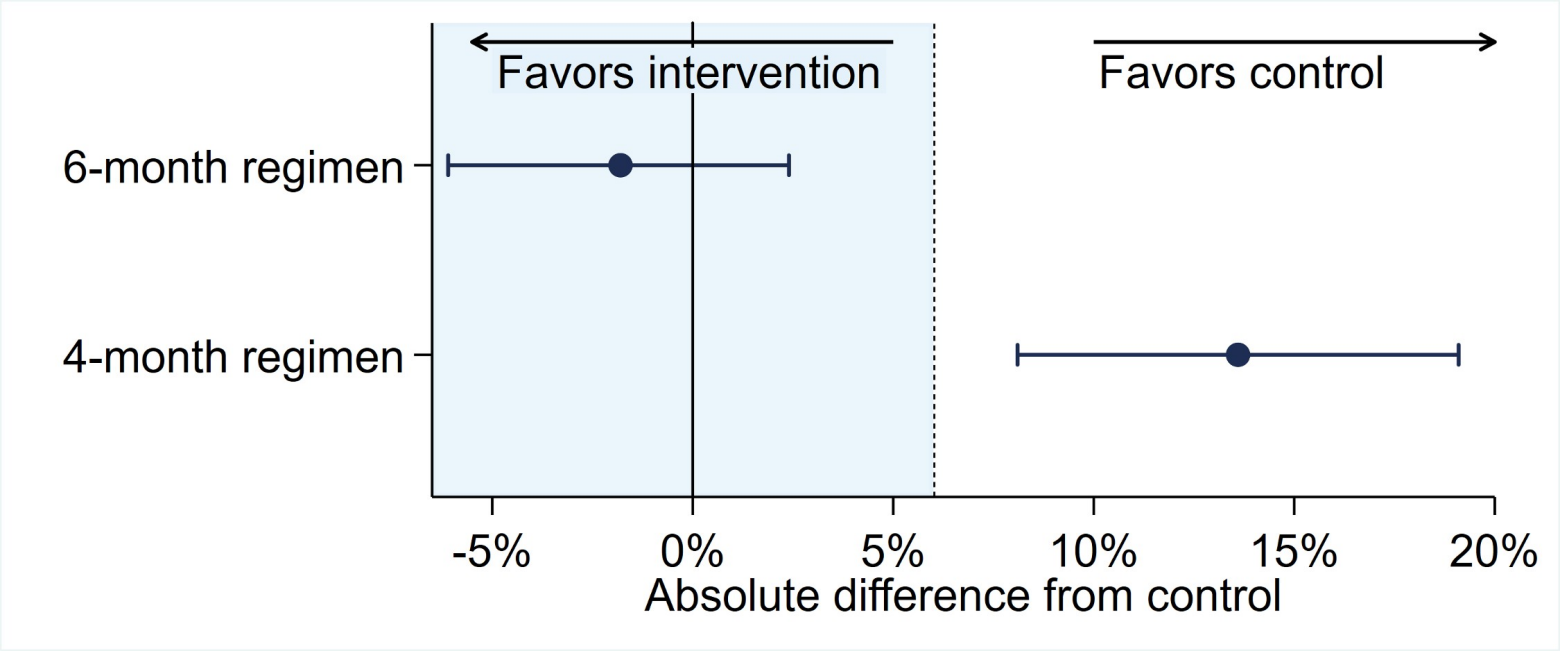
Illustration of the results of a noninferiority trial with a 6% margin of noninferiority (represented by the shaded area to the left of 6%), data from the RIFAQUIN trial [5]. The findings for two drug regimens are illustrated by point estimates of efficacy surrounded by 90% confidence intervals from the per protocol analysis. In this example, the 6-month regimen is noninferior to control because the upper bound of the confidence interval is less than the 6% margin of noninferiority. However, the 4-month regimen is not noninferior to the control because the upper bound exceeds the margin of noninferiority.

Noninferiority trials are designed to evaluate whether a reduction in efficacy with the intervention as compared to the control does not exceed a prespecified threshold. The prespecified difference is denoted as the noninferiority margin (see **Fig 1** for an illustration of the results of a noninferiority trial). The choice of the margin in trials of TB treatment regimens continues to be a major discussion issue. In order to have confidence that the new treatment is better than no treatment, it is accepted that the margin should be no larger, and considerably smaller, than the estimate of benefit of the chosen control as compared to no treatment. This effect is, however, estimated from historical data [41], and the statistical uncertainty of the estimate should be taken into account. For example, a somewhat conservative estimate of the success rate of standard therapy in DS-TB is around 85% [42], which, compared to the estimated 30% survival from untreated TB [43], gives an estimate of an absolute treatment effect of 55%. A declaration of noninferiority with a margin of 10% would therefore give confidence that more than 80% of this effect of the control is preserved, and 90% would be preserved with a margin of 5%.

Consideration of the expected benefits of the intervention shapes the final choice of margin. In TB, regimens that are shorter confer a direct benefit to patients and health systems. They are expected to result in better treatment adherence in addition to reduced patient and health system costs, although none of these can be easily measured in a clinical trial that is not close to usual practice. Anticipated reduction in toxicity is another factor that may influence the choice of margin; whether this is a consequence of the new treatment or not cannot be known until the trial has been completed. Collection of good safety data is critical to properly weigh the risks and benefits of an intervention. Combining efficacy and safety in a composite outcome or a formal risk–benefit scoring system [44] is useful to summarize this balance in a single measure. Such measures can, however, obscure differences between outcomes of varying severity. Papers summarizing the primary results of trials should, therefore, report safety and efficacy outcomes separately for others to make a judgment on the risk–benefit balance.

There has been a trend towards larger noninferiority margins in a number of recent protocols; this permits a reduction in sample size, resulting in a less expensive study and earlier completion, but leads to greater uncertainty as to the true efficacy of the regimen. Widening the margin increases the possibility that a substandard regimen could be accepted as a new gold standard, thereby increasing the risk of “biocreep,” whereby after several generations of noninferiority trials, considerably less effective regimens would become the standard of care simply because of the cumulative reduction in efficacy [45,46].

The intention-to-treat (ITT) analysis population includes all randomized participants in the groups to which they were allocated, irrespective of treatment received, loss to follow-up, or any protocol violations. In contrast, the per-protocol (PP) analysis population includes participants who achieve an adequate measure of compliance with the treatment and with the trial protocol [47]. The analysis population to be used in noninferiority trials has been the subject of recent debate since neither the ITT nor PP populations are free from bias, and reliance on either can increase the chance of falsely declaring noninferiority. In contrast to superiority trials, in which ITT is preferred because it provides “a secure foundation for statistical tests” [47], an ITT analysis can be biased towards noninferiority because of poor trial conduct diluting the treatment effect, whereas a PP analysis can also be biased in either direction when postrandomization exclusions from the analysis may be directly or indirectly related to treatment allocation.

While a PP population has been recommended for noninferiority trials in the past [45,47], its importance has been re-evaluated. FDA guidance no longer recommends PP (or as-treated) analysis [41], even though the 2010 draft guidance accommodated one. There are limitations in PP analyses, and proposed improvements include correcting for noncompliance and dependent censoring using inverse probability weighting [48]. Current guidance suggests, instead, to focus on ensuring trial quality to reduce the bias in the ITT analysis; consideration is also given to multiple imputation as a way to counter bias due to attrition [41]. The 2017 addendum (“estimands and sensitivity analysis in clinical trials” [49]) to the 1998 International Council for Harmonization of Technical Requirements for Pharmaceuticals for Human Use (ICH) E9 document (“statistical principles for clinical trials” [47]) goes beyond just specification of an analysis population by recommending the use of an estimand as a framework for aligning the target and method of estimation of a treatment effect with the objectives of the clinical trial. The estimand “defines in detail what needs to be estimated to address a specific scientific question of interest” [49] and includes four attributes: the population, the endpoint, the specification of how to account for non-endpoint intercurrent events, and the population-level summary. Practically, defining the primary estimand(s) of interest in the protocol before the trial starts promotes clarity and coherence in how a treatment effect is estimated and how it links back to the trial objective.

A further recent innovation has been the application of Bayesian methodology to the interpretation of noninferiority trials. Presentation of the results as a simple binary statement as to whether or not noninferiority has been achieved is of limited value because it gives no indication as to how close in efficacy the intervention is likely to be to the control and places undue emphasis on the often arbitrary noninferiority margin. A much more informative approach is to use a Bayesian analysis to provide the probability that the difference is less than some given percentage, say 5% [50].

## Role of adaptive trial designs

An adaptive clinical trial permits changes to various trial design features after trial initiation in response to accruing data [51]. Although potential changes must be prespecified in the protocol so as not to undermine trial validity and integrity, adaptive trial designs are nevertheless useful to account for uncertainty when a trial starts or for anticipating potential landscape changes that may occur during the course of the trial. Most common are (i) the inclusion of interim analyses that permit early stopping for overwhelming efficacy or lack of benefit when evidence is sufficiently compelling with a smaller sample size than anticipated, and (ii) sample size re-estimation during recruitment using a preplanned algorithm to ensure that the final size will be adequate to answer the research question (particularly relevant when there is uncertainty in the efficacy of the control arm).

When there are many potential combination regimens that might be considered for evaluation, one might consider designs that select among multiple regimens either by stopping recruitment to poorly performing arms after fixed-interval interim analyses (an example being the multiarm multistage [MAMS] design [52,53]) or by adjusting randomization probabilities to enroll more patients in more promising arms (Bayesian adaptive randomization [35]). When the toxicity of a regimen is unknown, one might consider designs in which the eligibility criteria are widened during the trial as more safety data accrue. This can, for instance, be performed by starting to recruit patients with extensively drug-resistant TB (XDR-TB) because few treatment options are available, then expanding to multidrug-resistant TB (MDR-TB) and DS-TB if safety thresholds are reached. Adaptations can, however, introduce bias in the estimate of treatment effect or inflate the probability of a false positive result (type I error rate). For example, in a two-stage multiarm trial in which only the intervention with the highest efficacy in the first stage is taken forward to the second stage, the uncorrected estimate of efficacy for this intervention at the end of the trial will be markedly biased and higher than the true efficacy [54]. Thorough understanding of these aspects is critical for regulatory approval and consideration in establishing WHO guidelines.

## Strategy trials incorporating treatment stratification

Treatment stratification, the process of splitting a patient population into a small number of groups who receive different treatments for the same disease based on a predictive biomarker, is being widely studied in other disease areas [55–57]. In TB, it has long been recognized that disease prognosis is affected by certain baseline factors such as pretreatment extent of cavitation and viable counts of TB bacteria [58]. Wallace Fox sowed the seeds of stratified medicine in 1981 [59] by noting that good prognostic factors could be used to tailor treatment duration. The first trial incorporating treatment stratification had an enrichment design (one in which eligibility criteria are restricted to or enriched for a particular subgroup of participants) that evaluated a 4-month regimen with no new drugs in patients with noncavitary disease and culture negativity at 2 months. Before recruitment finished, the trial was stopped by the safety monitoring committee because of an apparent increased risk for relapse in the 4-month arm [7]. However, the completion of several large multicenter randomized trials in DS-TB showed that a 4-month fluoroquinolone-based regimen may well be adequate for patients with noncavitary disease [60]. Subsequent analyses describing an algorithm to more precisely identify subgroups of patients with lower or higher risk of failure and relapse [61] have provided important evidence to support the evaluation of treatment stratification in TB trials. These data are only from rifampicin-containing regimens for DS-TB to date. Nevertheless, the principles are likely also relevant for DR-TB, for which reducing duration for patients who do not need it is even more important, given the high levels of toxicity of drugs and the longer duration of treatment [62].

Several trials are under development to evaluate new treatment strategies to assess different durations, drug combinations, or drug dosages according to patient risk factors [63]. These predictive biomarker validation trials are designed to “confirm” a stratification algorithm in a randomized comparison against the standard-of-care strategy of a fixed duration regimen for all patients [64]. They are distinct from more exploratory trials designed to “learn” or develop and optimize the stratification algorithm [55]. Such trials tend to be smaller or have highly adaptive designs and are also important to incorporate newer biomarkers into the stratification algorithms, often to be evaluated in a subsequent larger confirmative trial. With appropriate stratification, it is expected that it may be possible to target treatment strategies with superior efficacy to standard of care, thereby avoiding many of the pitfalls of noninferiority trials.

## The need for more pragmatic trials

In general, trials can be classified as explanatory (with the objective of evaluating the benefit an intervention produces under ideal conditions, i.e., efficacy) or pragmatic (with the objective of evaluating the benefit the treatment produces in routine clinical practice, i.e., effectiveness) [65,66], although this is more of a continuum than a dichotomy [67]. Trials that are more explanatory are needed to understand the efficacy and safety of a new drug under conditions as ideal as possible. However, the context in which an explanatory trial is conducted can be so far removed from routine practice that the results cannot readily be assumed to be transferable to clinical care. This is particularly the case when there are considerable changes in the landscape, as has been seen in DR-TB. The current acceptance of bedaquiline as a safe and efficacious drug in the treatment of MDR-TB is due less to the pivotal phase II background regimen study [40], which initially led to WHO guidelines recommending bedaquiline only under certain conditions [68], than to the extensive nonrandomized data gathered outside of a trial setting [15], mostly under program conditions. These programmatic data were influential in bedaquiline becoming one of the three priority medicines in the revised 2018 WHO guidelines for DR-TB [26], albeit based on low-quality evidence [69]. This reflects the way in which, in the absence of pragmatic trials, WHO guidelines have been based almost exclusively on observational data that yield conditional recommendations based on low-quality evidence.

Pragmatic randomized trials with broader eligibility criteria, greater geographical spread, use of programmatically relevant primary endpoints, use of best available standard of care as control (see **Table 2**), and delivery and adherence strategies that are closer to “real-life” conditions greatly increase generalizability of the results and lead to faster and more evidence-based changes to policy and practice. Such pragmatic trials have been necessary in evaluating effective treatment strategies for HIV using previously licensed drugs (the START trial [70], for example), and they will also be needed in TB. Pragmatic trials can also be embedded within implementation programs to evaluate population-level effects of an intervention, an example being the XTEND study, which was designed to evaluate the effect of the GeneXpert MTB/RIF during implementation in South Africa [71]. Clearly, no single trial can address all relevant questions about a given therapeutic intervention at one time, and pragmatic trials will be invaluable for public health and policy decision-making purposes.

## Conclusions

Just over 10 years ago, calls to action were published for innovations in drug development, capacity building for TB trials, and execution of clinical trials of treatment for DR-TB [72–74]. Since November, 2007, 538 TB clinical trials have been posted on clinicaltrials.gov; 27 (5%) of these have been for DR-TB. Between 1997 and 2007, these numbers were 127 and 4 (3%), respectively. Although the objectives and quality of these trials vary hugely, these raw numbers suggest that some progress has been made in clinical trial conduct.

The present review comes at a time when new drugs, new diagnostics, and new methods make possible real transformation in TB treatment. Today, there are 8 and 6 new compounds known to be in phase I and phase II clinical development, respectively (https://www.newtbdrugs.org/pipeline/clinical), with many more in preclinical development. The advances in clinical trial methodology that have been mentioned above alongside the promise of a variety of host-directed therapies [75] contrast starkly with the relative stagnation in treatment of DS- and DR-TB since the 1990s. The delivery of new regimens to patients demands nimbleness in an endeavor that is long and cumbersome. Trials must be designed and implemented in such a way that their relevance persists through completion. Careful choices of trial design, comparator, sample size, biomarker stratification, estimands, analysis population(s), and noninferiority margin are critical from the outset. Changes in some of these characteristics after trial initiation—through predefined adaptation and protocol amendments—must also be entertained. Continued weighing of implications for time, cost, interpretation, and impact on practice is essential; whether the trial is primarily explanatory or pragmatic is a decision based on the balance among these competing priorities for any given trial. Transparency around assumptions and factors influencing decision making is critical to interpretation by guidance developers, practitioners, and patients. Consultation with external experts, including community advisory boards, can facilitate this transparency.

In conclusion, we strongly believe that TB treatment trials today should favor innovative approaches that are able to produce high-quality evidence for high-quality, patient-centered care that can be made accessible to all 10 million new TB patients, including the half-million with DR-TB, each year.

## Abbreviations

DR-TB: drug-resistant TB
DS-TB: drug-sensitive TB
DSMB: data and safety monitoring board
EMA: European Medicines Agency
FDA: Food and Drug Administration
GCP: Good Clinical Practice
ICH: International Council for Harmonization of Technical Requirements for Pharmaceuticals for Human Use
ITT: intention to treat
MAMS: multiarm multistage
MDR-TB: multidrug-resistant TB
MRC: Medical Research Council
PP: per protocol
RR-TB: rifampicin-resistant TB
TB: tuberculosis
UN: United Nations
WHO: World Health Organization
XDR-TB: extensively drug-resistant TB
